# Advanced Imaging and Receipt of Guideline Concordant Care in Women with Early Stage Breast Cancer

**DOI:** 10.1155/2016/2182985

**Published:** 2016-07-25

**Authors:** Elizabeth Trice Loggers, Diana S. M. Buist, Laura S. Gold, Steven Zeliadt, Rachel Hunter Merrill, Ruth Etzioni, Scott D. Ramsey, Sean D. Sullivan, Larry Kessler

**Affiliations:** ^1^Clinical Research Division, Fred Hutchinson Cancer Research Center, Seattle, WA 98109, USA; ^2^Group Health Research Institute, Group Health Cooperative, Seattle, WA 98101, USA; ^3^Public Health Sciences, Fred Hutchinson Cancer Research Center, Seattle, WA 98109, USA; ^4^Department of Health Services, School of Public Health, University of Washington, Seattle, WA 98195, USA; ^5^Pharmaceutical Outcomes Research and Policy Program, School of Pharmacy, University of Washington, Seattle, WA 98195, USA; ^6^Health Services Research and Development, Department of Veterans Affairs, Puget Sound Health Care System, Seattle, WA 98174, USA

## Abstract

*Objective.* It is unknown whether advanced imaging (AI) is associated with higher quality breast cancer (BC) care.* Materials and Methods.* Claims and Surveillance Epidemiology and End Results data were linked for women diagnosed with incident stage I-III BC between 2002 and 2008 in western Washington State. We examined receipt of preoperative breast magnetic resonance imaging (MRI) or AI (defined as computed tomography [CT]/positron emission tomography [PET]/PET/CT) versus mammogram and/or ultrasound (M-US) alone and receipt of guideline concordant care (GCC) using multivariable logistic regression.* Results.* Of 5247 women, 67% received M-US, 23% MRI, 8% CT, and 3% PET/PET-CT. In 2002, 5% received MRI and 5% AI compared to 45% and 12%, respectively, in 2008. 79% received GCC, but GCC declined over time and was associated with younger age, urban residence, less comorbidity, shorter time from diagnosis to surgery, and earlier year of diagnosis. Breast MRI was associated with GCC for lumpectomy plus radiation therapy (RT) (OR 1.55, 95% CI 1.08–2.26, and *p* = 0.02) and AI was associated with GCC for adjuvant chemotherapy for estrogen-receptor positive (ER+) BC (OR 1.74, 95% CI 1.17–2.59, and *p* = 0.01).* Conclusion.* GCC was associated with prior receipt of breast MRI and AI for lumpectomy plus RT and adjuvant chemotherapy for ER+ BC, respectively.

## 1. Introduction

In the last 10 years, use of advanced imaging (defined here as magnetic resonance imaging (MRI), computed tomography (CT), or positron emission tomography (PET)) has become the single fastest growing health care service, outstripping all other tests and procedures [1–6]. This trend may be particularly pronounced for cancer patients, who typically receive multiple scans during all phases of cancer care (diagnosis, treatment, surveillance, and end of life) [7–10]. For example, PET scanning rates rose faster than any other imaging modality among both Medicare beneficiaries and HMO enrollees of all ages with cancer [7–9]. Despite this rapid rate of growth, the relationship between advanced imaging and health outcomes is relatively unknown.

Amongst the imaging modalities, receipt of breast magnetic resonance imaging (MRI) in diagnosis and perioperative planning for breast cancer has received significant attention [11–17]. While breast MRI identifies occult cancers in individuals not identified as cases by clinical breast exam, mammography, or ultrasound [13, 18], there is no evidence that identification of clinically or mammographically occult tumors improves recurrence or survival outcomes [17–19]. Furthermore, two recent prospective randomized controlled trials of preoperative breast MRI showed no reduction in reexcision rates and a trend toward longer time to definitive therapy [20, 21]. Findings such as these have led some to call for greater evidence linking receipt of advanced imaging to improvements in health care quality or outcomes [17, 19].

As there is evidence supporting higher quality outcomes among individuals receiving guideline concordant care (GCC) [20–24], one approach to assessing the clinical impact of advanced imaging is to examine whether receipt of preoperative breast MRI (or other advanced imaging) is associated with receipt of guideline concordant care (GCC) for breast cancer. While this relationship would not necessarily be causal, learning more about the association between receipt of advanced imaging and GCC could be informative to patients, insurers, and policy makers who struggle with defining “appropriateness” with respect to imaging studies. Therefore this study was designed to investigate associations between receipt of advanced imaging and GCC for breast cancer, focusing on well-known and accepted guidelines including, for example, radiation following lumpectomy and adjuvant chemotherapy for estrogen-receptor-positive disease.

## 2. Materials and Methods

This study was approved by the Institutional Review Boards at the participating institutions.

### 2.1. Study Population

We linked health plan enrollment and utilization files from three commercial health plans (Regence Blue Shield, Premera Blue Cross, and Uniform Medical Plan), one mixed-model health delivery system (Group Health Cooperative) and two publically funded health care programs (Medicaid and Medicare) with the western Washington Surveillance Epidemiology and End Results registry [25]. The linkage identified all adult (age: 18 years or older) females diagnosed with an incident invasive breast carcinoma between 2002 and 2008.

**Figure 1:**
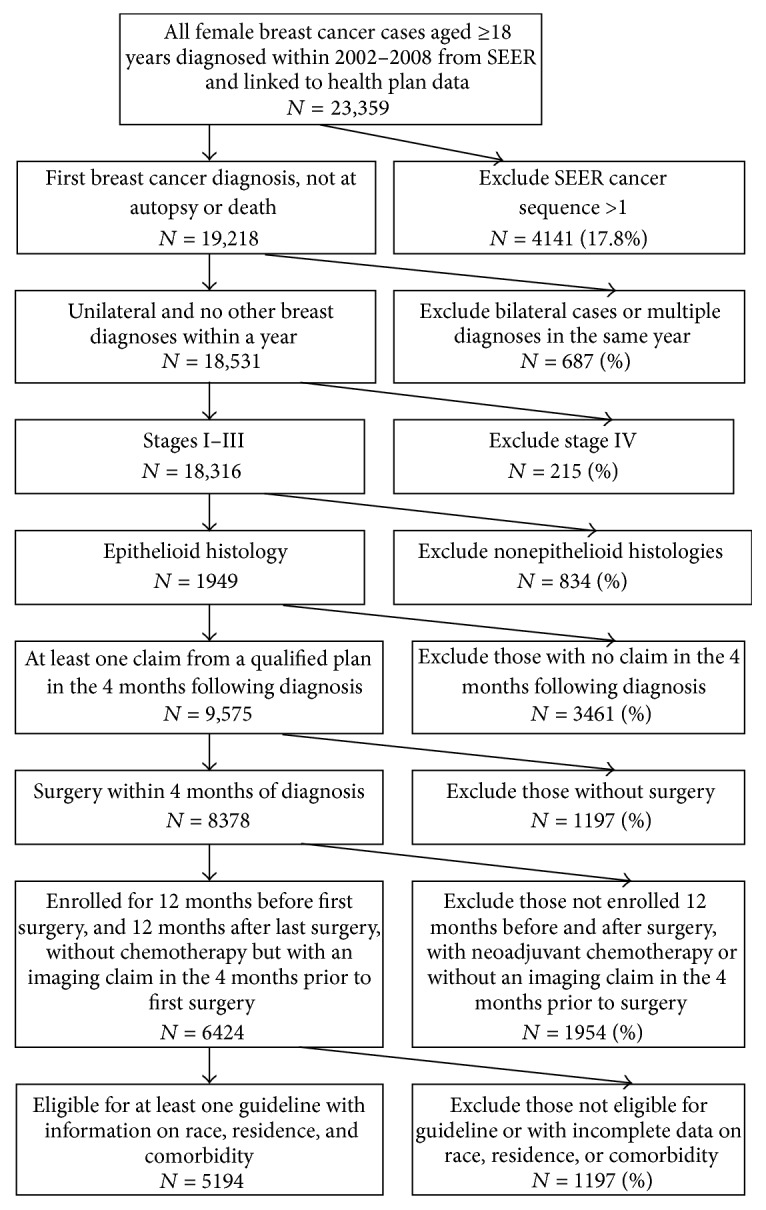
Study CONSORT diagram.

**Figure 2:**
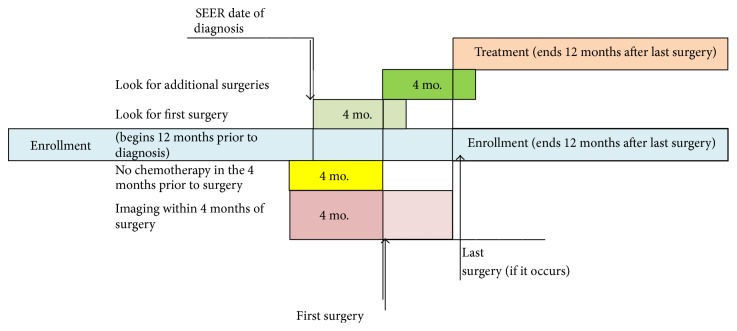
Study schema for identifying event windows.

We then excluded women who (1) had been diagnosed at autopsy or death, (2) had any additional breast cancer diagnosed within one year of the index diagnosis or bilateral cancer, (3) had stage IV cancer [26], (4) had a nonepithelial histology, (5) had no health plan claim in the 4 months following diagnosis, (6) received no surgery for treatment, (7) were not enrolled (gaps in coverage of no more than 60 days) for at least 12 months before and after surgery (unless they died), with neoadjuvant chemotherapy or without an imaging claim in the 4 months before surgery, and/or (8) were not eligible for at least one NCCN guideline and had no available information on race, comorbidity, or residence (Figures 1 and 2). Women enrolled in more than one health plan in the sixty days before and after diagnosis (*n* = 397) were also excluded from the analytic model to allow comparison among women enrolled in only one type of health plan.

We required women to have received their first breast surgery (either mastectomy or lumpectomy) within 4 months of the SEER date of diagnosis, since women who do not receive definitive surgery are substantially different. To be guideline concordant, care had to occur within one year of surgery; however, some women receive mastectomy or further surgery for positive margins following an initial lumpectomy. To address this, we looked up to four months after a lumpectomy for evidence of a mastectomy or further surgery and used the date of the final surgery (also referred to as the “last” surgery date) as the beginning of the period in which to determine whether guideline concordant care had been received (Figure 2). Women receiving a mastectomy after an initial lumpectomy were classified as receiving a mastectomy (along with all other women who received an initial mastectomy without a preceding lumpectomy).

### 2.2. Identification and Classification of Imaging

We focused on imaging used for staging and treatment planning before definitive surgery for breast cancer; thus, imaging was anchored to surgery date rather than diagnosis date (Figure 2). We included all imaging occurring for four months prior to the first surgery through the last surgery (when additional surgery occurred). We used the presence of at least one Current Procedural Terminology (CPT) code to identify mammograms with or without ultrasound, MRI, CT, and PET/PET-CT. Women were classified into mutually exclusive and hierarchical categories as receiving (1)* no imaging besides mammography or ultrasound*, (2)* breast MRI*, and (3)* other advanced imaging* (CT and/or PET/PET-CT). Finally, we present CT separate from PET/PET-CT in Table 1 to demonstrate differences in use; however for the multivariable analysis in which guideline concordant care is the outcome, we included CT with PET/PET-CT as described above.

### 2.3. Defining Treatment and Guideline Concordant Care

We used CPT codes to identify surgery (mastectomy and lumpectomy) and radiation CPT and/or Healthcare Common Procedural Coding System (HCPCS) J-code to identify chemotherapy receipt within one year of the last surgery. Tumor characteristics (i.e., size and receptor status) and lymph node status were determined via SEER data.

Definitions of GCC were derived from National Comprehensive Cancer Network (NCCN) published guidelines [27]. To increase the likelihood that there was wide clinical support (i.e., awareness of and agreement on the importance of the guidelines in the clinical care of early stage breast cancer) for the inclusion of our study guidelines throughout the study period, we selected guidelines based on two criteria: (1) data supporting selection of the guideline had existed for at least 5 years before the start of the study period (2002) and (2) each guideline was present in the NCCN guidelines for the entire study period. Using these criteria, four guidelines were operationalized for this study (*N* received/*N* eligible for measure): (1) adjuvant radiation receipt following lumpectomy (*Radiation following Lumpectomy*) (3,736/4085, 91.5%), (2) radiation receipt following mastectomy if 4 or more axillary lymph nodes were positive (stage III disease) (*Radiation following Mastectomy for Stage III*) (285/321, 88.8%), (3) adjuvant chemotherapy received for women with estrogen-receptor positive tumors with at least one positive lymph node (*Chemotherapy for ER+ Disease*) (929/1,453, 63.9%), and (4) adjuvant chemotherapy received for women with ER− tumors, >1 centimeter or with at least one positive lymph node (*Chemotherapy for ER*−* Disease*) (578/816, 70.8%). We did not examine guidelines associated with adjuvant endocrine therapy receipt because of the inability to access these data through claims.

In order to assess guideline concordance, we first assessed whether each woman was eligible for the respective guideline. For example, surgical categories were mutually exclusive, so women who were eligible for the* Radiation following Lumpectomy* guideline were not eligible for the* Mastectomy following Radiation* guideline; similarly women eligible for the* Chemotherapy for ER+ Disease* guideline were not eligible for the* Chemotherapy for ER− Disease* guideline. We defined concordance for women for each eligible guideline.

### 2.4. Covariates

We considered sociodemographic variables, comorbidity, health plan characteristics, diagnosis year, and time from diagnosis to definitive surgery as potential confounding variables. Using SEER, we examined diagnosis year, age, race/ethnicity, urban or rural residence (based on her zip code diagnosis using the 2000 US Census [28]), and median household income (US Census tract at diagnosis). Comorbidity was assessed using claims data from health plans from one year to 30 days before the first surgical procedure [29]. An exception was made for determining comorbidity in Medicaid patients due to differences in enrollment patterns; we looked from four months to one year prior to the first surgery depending upon available enrollment data. Payer characteristics included health plan type (e.g., fee for service, managed care, Medicaid, and Medicare). Time from diagnosis to definitive surgery was calculated using the SEER date of diagnosis and the claims date of last surgery. Stage and/or tumor characteristics and estrogen receptor status are not included in the model as possible confounding variables because they were used in the definition of the GCC as described above.

### 2.5. Statistical Analysis

We performed univariate logistic regressions to identify factors associated with each imaging category. We used results of these analyses to construct multivariable logistic regression models comparing the breast MRI and other advanced imaging receipt to mammogram ± ultrasound. Because of the small number of women eligible for receipt of some guidelines and the relatively large number of independent variables selected* a priori*, some variable categories were collapsed (i.e., race and time to definitive surgery) or entered into the model as continuous variables (i.e., age, comorbidity, and income).

## 3. Results

The final sample included 5,247 subjects, of whom 67% received only mammogram and/or ultrasound, 23% received breast MRI, 8% received CT, and 3% received PET/PET-CT before definitive surgery (see Table 1). In 2002, 89% of women received a mammogram and/or US, while 5% of women received an MRI and 5% received advanced imaging; by 2008 the percentages changed to 42%, 45%, and 12%, respectively. Younger women were more likely to have received breast MRI and PET/PET-CT. Women residing in urban locations and who had higher incomes and fewer comorbidities were more likely to have received breast MRI, as were women diagnosed in later years of the study.

Most women (79%) received all GCC for which they were eligible (Table 2); guideline concordance for women receiving chemotherapy was lower than for other guidelines (64% ER+ and 71% ER−). In the model examining associations between overall GCC and receipt of advanced imaging, older age, living in a nonurban setting, increasing comorbidity, increasing time from diagnosis to surgery, and later year of cancer diagnosis were negatively associated with receipt of all GCC for which a woman was eligible (Table 3). The likelihood of receiving GCC appeared to decline over time (OR 0.74, 95% CI 0.55–0.99, and *p* = 0.04 in 2004; OR 0.66, 95% CI 0.50–0.89, and *p* = 0.01 in 2008).

MRI receipt was associated with increased odds of receiving radiation following a lumpectomy (OR 1.55; 95% CI 1.08–2.26; *p* = 0.02), but not with advanced imagining (Table 4). However, advanced imaging receipt was associated with increased odds that a woman would receive adjuvant chemotherapy for ER-positive disease (OR 1.74; 95% CI 1.17–2.59; *p* = 0.01). No other statistically significant associations were found between MRI or advanced imaging receipt and GCC.

## 4. Discussion

Our study is among the first to investigate the relationship between advanced imaging and receipt of guideline concordant care. We found a rapid rate of increase in breast MRI and advanced imaging and a relatively high percentage of women who received all guideline concordant care. MRI was associated with an increased likelihood of receiving radiation following lumpectomy and advanced imaging was associated with an increased likelihood of receiving chemotherapy for ER-positive disease.

In light of our results, advanced imaging may be associated with temporal trends in (increasing) aggressiveness of care or unmeasured clinical or patient-related factors that are also associated with guideline concordant care. For example, clinicians may both scan and treat more aggressively patients they view as having higher risk disease. Patient preference and access may also play a role in both receipt of advanced imaging and guideline concordant care, both of which we were unable to account for in this study. Factors associated with a greater likelihood of achieving all guideline concordant care included younger age, urban residence, less comorbid disease, and shorter time from diagnosis to definitive surgery, consistent with prior studies [30–33]. Some of these factors have been previously associated with advanced imaging by our team [25]. Finally, the lack of significance for breast MRI and advanced imaging in relation to some study guidelines may be due to a lack of power (*N* = 321 eligible women for the* Radiation following Mastectomy* guideline and *N* = 816 eligible women for the* Chemotherapy for ER− Disease* guideline).

Of interest in this study is the observation that the proportion of women receiving guideline concordant care may be declining over time. We are unaware of any other studies that have reported similar results. This finding may be incidental or there may have been other guidelines or clinical practices that were not included in this study that affected care during the study period. Also, while we did not evaluate this hypothesis, the reduction in guideline concordant care might be due to increasing rates of lymph node dissection and receptor-status testing over the study period, which may lead to more women being eligible for more guidelines and, in turn, more opportunities to fail to receive “all” guideline concordant care. Alternatively, reductions in the rate of receipt of all guideline concordant care may be due to greater awareness on the part of clinicians and/or patients of the long-term negative consequences of adjuvant radiation [34–36] and chemotherapy [37–40] and in some cases the potentially limited benefit of adjuvant chemotherapy [33, 41–43], leading to lower rates of use despite guideline recommendations.

While this study is the first to examine associations between advanced imaging and guidelines concordant care in a large, population-based setting, it does have limitations. This study cannot address why a woman did not receive guideline concordant care. It is possible that some women did not receive guideline recommended care* appropriately* due to complications from earlier treatment, comorbidities, personal preference, or other reasons. We were unable to include all guidelines, such as adjuvant endocrine therapy receipt, given limitations in claims data. Indication for the exam was unavailable; it is possible that women received imaging for reasons other than their cancer diagnosis. In addition, our study includes women over the age of 70, for whom the value of adjuvant chemotherapy is less clear.

In conclusion, this study characterizes the relationship between MRI and advanced imaging receipt and overall quality of care for women with early stage breast cancer, both topics of increasing importance as the goal of achieving high-quality care at the lowest cost is pursued. Future research should investigate the clinical rationale and patient factors influencing MRI and advanced imaging receipt to better inform the relationship between advanced imaging and receipt of high-quality, guideline concordant breast cancer care.

## Figures and Tables

**Table 1:** Characteristics of 5,247 women receiving different types of imaging (in mutually exclusive categories^1^) prior to definitive surgery for early stage breast cancer.

	Mammogram/ultrasound^1^	Breast MRI^2^	CT^3^	PET/PET-CT^4^	Total (%)
	(*n* = 3,525, 67.2%)	(*n* = 1,190, 22.7%)	(*n* = 394, 7.5%)	(*n* = 138, 2.6%)	(*n* = 5,247)
*Age at diagnosis (mean ± standard deviation)*	68.2 ± 12.2	61.1 ± 12.0	64.4 ± 13.2	59.8 ± 13.2	66.1 ± 12.7

*Age at diagnosis*					
≤50 years^*∗*^	290 (8.2%)	218 (18.3%)	62 (15.7%)	38 (13.8%)	599 (11.4%)
51–64 years	946 (26.8%)	484 (40.7%)	113 (28.7%)	62 (44.9%)	1,605 (30.6%)
65–70 years	677 (19.2%)	191 (16.1%)	83 (21.1%)	20 (14.5%)	971 (18.5%)
≥71 years^*∗*^	1,612 (47.1%)	297 (24.9%)	136 (26.5%)	18 (19.5%)	2,072 (39.5%)

*Race*					
White	3,267 (92.7%)	1,107 (93.0%)	359 (91.1%)	122 (88.4%)	4,855 (92.5%)
Non-White^*∗*^	258 (7.3%)	83 (7.0%)	35 (8.9%)	16 (11.6%)	392 (7.5%)

*Race/ethnicity*					
White non-Hispanic	3,232 (91.7%)	1,098 (92.3%)	353 (89.6%)	121 (87.7%)	4,804 (91.6%)
Non-White and/or Hispanic^*∗*^	293 (8.3%)	92 (7.7%)	41 (10.4%)	17 (12.3%)	443 (8.4%)

*Rural/urban residence*					
Urban	3,101 (88.0%)	1,099 (92.4%)	345 (87.6%)	127 (92.0%)	4,672 (89.0%)
Rural	424 (12.0%)	91 (7.6%)	49 (12.4%)	11 (8.0%)	575 (11.0%)

*Health insurer type* ^5^					
Medicare	1,898 (53.8%)	473 (39.7%)	210 (53.3%)	50 (36.2%)	2,631 (50.1%)
Fee for service/managed care^*∗*^	1612 (45.7%)	717 (60.3%)	169 (42.9%)	71 (51.4%)	2401 (45.8%)
Medicaid^6^	116 (3.3%)	44 (3.7%)	35 (8.9%)	20 (14.5%)	215 (4.1%)

*2000 Census tract median income*					
*Median income (± standard deviation)*	48,819 ± 15,626	53,972 ± 16,619	50,570 ± 19,283	49,451 ± 18,460	50,250 ± 16,338
<$41,100	944 (26.8%)	219 (18.4%)	105 (26.6%)	37 (26.8%)	1,305 (24.9%)
$41,101–50,400	979 (27.8%)	261 (21.9%)	87 (22.1%)	36 (26.1%)	1,363 (26.0%)
$50,401–61,700	845 (24.0%)	340 (28.6%)	97 (24.6%)	28 (20.2%)	1,310 (25.0%)
$61,701+	757 (21.5%)	370 (31.1%)	105 (26.6%)	37 (26.8%)	1,269 (24.2%)

*Year of diagnosis*					
2002–2005^*∗*^	2204 (62.5%)	366 (30.7%)	201 (51.1%)	19 (13.8%)	2790 (53.2%)
2006	541 (15.3%)	206 (17.3%)	62 (15.7%)	27 (19.6%)	836 (15.9%)
2007	447 (12.7%)	262 (22.0%)	75 (19.0%)	50 (36.2%)	834 (15.9%)
2008	333 (9.5%)	356 (30.0%)	56 (14.2%)	42 (30.4%)	787 (15.0%)

*Comorbidity (CITE)*					
*0*	2,668 (75.7%)	1,032 (86.7%)	309 (78.4%)	106 (76.8%)	4,115 (78.4%)
≥*1* ^*∗*^	857 (24.3%)	158 (13.2%)	85 (21.6%)	32 (23.2%)	1132 (21.6%)

*Time from diagnosis to definitive surgery*					
Mean days ± standard deviation	26.1 ± 17.5	31.8 ± 18.7	32.1 ± 20.0	38.4 ± 24.5	28.1 ± 18.5

*Time from diagnosis to definitive surgery*					
0–14 days^*∗*^	936 (26.5%)	161 (13.5%)	62 (15.7%)	16 (11.6%)	1175 (22.4%)
15–30 days	1,394 (39.5%)	495 (41.6%)	147 (37.3%)	48 (34.8%)	2,084 (39.7%)
31–60 days	1,043 (29.6%)	444 (37.3%)	155 (39.3%)	52 (37.7%)	1,694 (32.3%)
61 or more days	152 (4.3%)	90 (7.6%)	30 (7.6%)	22 (5.9%)	294 (0.1%)

^*∗*^Due to small cells, some rows have been suppressed by aggregating across other rows.

^1^Women may receive more than one type of imaging but are categorized only once in the table by the highest intensity of imaging. The hierarchy is PET/PET-CT > CT > MRI > mammogram with or without ultrasound (as the base category).

^2^MRI: magnetic resonance imaging.

^3^CT: computed tomography.

^4^PET/PET-CT: positron emission tomography.

^5^Patients may be included in more than one health insurance category if dual-enrolled. This categorization allows nonqualifying enrollment in the period ±60 days of diagnosis.

^6^The possibility of being insufficient claims or Medicaid with limited enrollment prior to diagnosis.

**Table 2:** Distribution of covariates by receipt of all guideline concordant care for which a woman was eligible and by individual guideline, for women with early stage breast cancer.

Variable	All guideline concordant care	Radiation following Lumpectomy	Radiation following Mastectomy	Chemotherapy for ER-positive Disease^1^	Chemotherapy for ER-negative Disease^2^
*N*	5,247	4,085	321	1,453	816

Concordant					
Yes	4,168 (79.4%)	3,736 (91.5%)	285 (88.8%)	929 (63.9%)	578 (70.8%)
No	1,079 (20.6%)	349 (8.5%)	36 (11.2%)	524 (36.1%)	238 (29.2%)

Imaging					
Mammogram/US	3,525 (67.2%)	2,888 (70.7%)	148 (46.1%)	858 (59.1%)	542 (66.4%)
Breast MRI	1,190 (22.7%)	921 (22.5%)	82 (25.5%)	350 (24.1%)	145 (17.8%)
PET/CT	532 (10.1%)	276 (6.8%)	91 (28.3%)	245 (16.9%)	129 (15.8%)

Age^3^ (mean years ± standard deviation, range)	66.1 ± 12.7 (26–103)	66.4 ± 12.2 (26–103)	64.5 ± 13.9 (32–92)	64.3 ± 13.0 (30–93)	64.2 ± 13.8 (26–96)

Race (White non-Hispanic)					
Yes	4,804 (91.6%)	3,773 (92.4%)	284 (88.5%)	1,328 (91.4%)	718 (88.0%)
No	443 (8.4%)	312 (7.6%)	37 (11.5%)	125 (8.6%)	98 (12%)

Rural/urban residence					
Urban	4,672 (89.0%)	3,632 (88.9%)	291 (90.7%)	1,295 (89.1%)	714 (87.5%)
Large	360 (6.9%)	283 (6.9%)	18 (5.6%)	104 (7.2%)	61 (7.5%)
Small	215 (4.1%)	170 (2.4%)	12 (3.7%)	54 (3.7%)	41 (5.0%)

Health insurer type					
Medicare	2,498 (47.6%)	1,981 (48.5%)	133 (41.4%)	661 (45.5%)	339 (41.5%)
Fee for service	1,059 (20.2%)	822 (20.1%)	61 (19.0%)	307 (21.0%)	177 (21.7%)
Managed care	1,334 (25.4%)	1,027 (25.1%)	97 (30.2%)	370 (25.5%)	233 (28.6%)
Medicaid	189 (3.6%)	125 (3.1%)	18 (5.6%)	72 (5.0%)	42 (5.1%)
Multiple	167 (3.2%)	130 (3.2%)	12 (3.7%)	43 (3.0%)	25 (3.1%)

Log of median income (mean ± standard deviation, range)	10.8 ± 0.3 (9.3–11.8 )	10.8 ± 0.3 (9.3–11.8)	10.8 ± 0.3 (9.6–11.8)	10.8 ± 0.3 (9.3–11.8)	10.8 ± 0.3 (9.3–11.8)

Year of diagnosis					
2002	622 (11.9%)	491 (12.0%)	40 (12.5%)	174 (12.0%)	87 (10.7%)
2003	663 (12.6%)	502 (12.3%)	43 (13.4%)	177 (12.2%)	113 (13.8%)
2004	673 (12.8%)	518 (12.7%)	46 (14.3%)	196 (13.5%)	114 (14.0%)
2005	832 (15.9%)	642 (15.7%)	51 (15.9%)	235 (16.2%)	129 (15.8%)
2006	836 (15.9%)	672 (16.5%)	45 (14.0%)	209 (14.4%)	128 (15.7%)
2007	834 (15.9%)	641 (15.7%)	58 (18.1%)	248 (17.1%)	126 (15.4%)
2008	787 (15.0%)	619 (15.2%)	38 (11.8%)	214 (14.7%)	119 (14.6%)

Comorbidity^4^ (mean ± standard deviation, range)	0.2 ± 0.4 (0–3.4)	0.1 ± 0.4 (0–3.4)	0.2 ± 0.4 (0–2.5)	0.2 ± 0.4 (0–3.3)	0.2 ± 0.4 (0–3.1)

Time from diagnosis to definitive surgery (days)					
≤60	4,683 (89.3%)	3,665 (89.7%)	286 (89.1%)	1,280 (88.1%)	740 (90.7%)
>60	564 (10.7%)	420 (10.3%)	35 (10.9%)	173 (11.9%)	76 (9.3%)

^1^ER+: estrogen receptor positive.

^2^ER−: estrogen receptor negative.

^3^Reported are counts for categorical variables and mean ± standard deviation (range as minimum–maximum) for continuous variables.

^4^Klabunde comorbidity index.

**Table 3:** Multivariable model results for receipt of all guideline concordant care for which a woman was eligible for 5194 women with early stage breast cancer, emphasizing the relationship between breast MRI and advanced imaging.

	Odds ratio	95% confidence intervals	*p* value
*Mammogram/ultrasound*	*Ref.*	—	—
Breast MRI	**1.21**	**(0.98, 1.51)**	**0.07**
CT, PET, and PET/CT	**0.87**	**(0.68, 1.12)**	**0.28**

Age	0.93	(0.91, 0.93)	<0.0001

White, non-Hispanic	Ref.	—	—
No	0.96	(0.73, 1.28)	0.78

Rural/urban residence (urban)	Ref.	—	—
Large rural	0.65	(0.50, 0.86)	0.001
Small rural/isolated	0.73	(0.52, 1.04)	0.08

Plan type (Medicare)	Ref.	—	—
Fee for service	1.07	(0.79, 1.44)	0.67
Managed care	1.08	(0.89, 1.32)	0.43
Medicaid	0.67	(0.41, 1.13)	0.12
Multiple	0.99	(0.66, 1.51)	0.95

Log median income	1.17	(0.91, 1.51)	0.22

Year of diagnosis	Ref.	—	—
2003	0.77	(0.58, 1.03)	0.07
2004	0.74	(0.55, 0.99)	0.04
2005	0.73	(0.55, 0.96)	0.03
2006	0.73	(0.55, 0.97)	0.03
2007	0.75	(0.56, 0.99)	0.05
2008	0.66	(0.50, 0.89)	0.01

Comorbidity^1^	0.65	(0.55, 0.77)	<0.0001

Diagnosis to definitive surgery			
≤60 days	Ref.	—	—
>60 days	0.65	(0.52, 0.81)	0.0001

MRI: magnetic resonance imaging.

CT: computed tomography.

PET: positron emission tomography.

^1^Klabunde comorbidity index.

**Table 4:** Multivariable results by guideline for the association between receipt of advanced imaging and receipt of guideline concordant care for women with early stage breast cancer controlling for confounding variables.

Guideline	Odds ratio	95% confidence interval	*p* value
*Radiation following Lumpectomy *(*N* = 4,085)			
Mammogram/ultrasound	Ref.	—	—
Breast MRI	1.55	(1.08, 2.26)	0.02
CT, PET, and PET/CT	1.02	(0.67, 1.63)	0.92

*Radiation following Mastectomy *(*N* = 321)			
Mammogram/ultrasound	Ref.	—	—
Breast MRI	0.53	(0.19, 1.53)	0.24
CT, PET, and PET/CT	1.08	(0.39, 3.17)	0.88

*Chemotherapy for ER+ Disease *(*N*= 1,453)			
Mammogram/ultrasound	Ref.	—	—
Breast MRI	1.37	(0.96, 1.96)	0.09
CT, PET, and PET/CT	1.74	(1.17, 2.59)	0.01

*Chemotherapy for ER− Disease *(*N* = 816)			
Mammogram/ultrasound	Ref.	—	—
Breast MRI	1.07	(0.58, 2.00)	0.83
CT, PET, and PET/CT	1.61	(0.89, 2.97)	0.12

ER+: estrogen receptor positive.

ER−: estrogen receptor negative.

MRI: magnetic resonance imaging.

CT: computed tomography.

PET: positron emission tomography.

Confounding variables included age, race, residence, plan type, income, year, comorbidity, and time from diagnosis to definitive surgery.
